# Model‐based image restoration targeting higher‐dose digital mammography: Impact on radiologists' detection of clustered microcalcifications

**DOI:** 10.1002/mp.70739

**Published:** 2026-10-26

**Authors:** Renann F. Brandão, Lucas R. Borges, Silvia M. P. S. Sabino, Renato F. Caron, Alessandro Foi, Predrag R. Bakic, Anders Tingberg, Marcelo A. C. Vieira

**Affiliations:** ^1^ Department of Electrical and Computer Engineering, São Carlos School of Engineering University of São Paulo São Carlos Brazil; ^2^ Real Time Tomography, LLC Villanova USA; ^3^ Barretos Cancer Hospital Pio XII Foundation Barretos Brazil; ^4^ Signal Processing Research Centre Tampere University Tampere Finland; ^5^ Department of Radiology University of Pennsylvania Philadelphia USA; ^6^ Department of Translational Medicine, Faculty of Medicine, Diagnostic Radiology Lund University Malmö Sweden; ^7^ Department of Translational Medicine, Faculty of Medicine, Medical Radiation Physics Lund University Malmö Sweden; ^8^ Department of Hematology, Oncology and Radiation Physics, Radiation Physics Skåne University Hospital Malmö Sweden

**Keywords:** denoising, digital mammography, human observer study, image restoration, microcalcification detectability, noise modeling

## Abstract

**Background:**

Image noise in digital mammography (DM) can obscure image details and reduce the detectability of clinically relevant findings such as microcalcifications.

**Purpose:**

To present and evaluate an extended model‐based (MB) image restoration pipeline for DM that incorporates quantum, electronic, and structural noise components, as well as spatial noise correlation, and to assess its effect on breast radiologists' detection of simulated microcalcification clusters in clinical mammograms.

**Methods:**

A total of 120 clinical DM images acquired using a GE Senographe Pristina system were used. Simulated microcalcification clusters were inserted into locally dense fibroglandular regions at controlled contrast levels. The restoration pipeline was configured to target the signal and noise characteristics predicted by the model for an acquisition at approximately twice the original radiation dose. Six breast radiologists with different levels of experience, classified as Junior or Senior, participated in two human observer experiments: (i) a pilot study using original clinical images with simulated clusters and a four‐alternative forced‐choice (4AFC) protocol combined with a three‐down‐one‐up (3D1U) staircase to estimate reader‐specific contrast detection thresholds and achieve comparable baseline task difficulty across readers; and (ii) a main 4AFC observer study using individualized datasets generated according to each reader's estimated contrast threshold, in which microcalcification detection performance was compared between the original and restored image conditions based on the percentage of correct responses (PC). Two generalized linear mixed models (GLMMs) were used: the first evaluated the effect of image condition as a fixed effect, with reader and case as crossed random intercepts, and the second evaluated the effect of reader experience on detection performance in the original image condition.

**Results:**

At the reader‐specific contrast thresholds estimated in the pilot study, the mean PC for the original images was 78.3%, within the prespecified target performance range. Mean PC increased to 84.3% for the restored images, corresponding to an absolute improvement of 6.0 percentage points. The primary GLMM indicated a significant restoration effect on detection performance (OR = 1.57, 95% CI: 1.15–2.14, p=0.005). Baseline mean PC was 78.7% for Junior readers and 78.0% for Senior readers, and the separate GLMM found no evidence of an experience‐group effect under the original image condition (p=0.858).

**Conclusions:**

The proposed extended MB restoration pipeline improved the detectability of simulated microcalcification clusters by breast radiologists under the controlled 4AFC conditions investigated. By targeting the signal and noise characteristics predicted by the model for an acquisition at approximately twice the original radiation dose without requiring additional radiation exposure, the method represents a promising approach for improving microcalcification detectability in DM.

## 1 INTRODUCTION

Digital mammography (DM) is one of the most widely used imaging modalities for breast cancer screening and plays a key role in the early detection of the disease.^1^, ^2^ Because DM uses ionizing radiation, patient exposure must be carefully optimized to achieve adequate image quality while minimizing radiation‐related risks.^3^, ^4^ For a fixed beam quality and acquisition configuration, reducing radiation dose increases quantum noise relative to the image signal and may impair the visibility of subtle findings.^5^ Conversely, increasing the dose can improve the signal‐to‐noise ratio but involves greater radiation exposure and an associated risk of radiation‐induced cancer.^6^, ^7^

Consequently, developing techniques that improve mammography image quality without increasing patient radiation exposure remains an important goal. Among these approaches, image restoration has emerged as a promising strategy for reducing image noise and improving the visibility of clinically relevant structures.^8^, ^9^, ^10^

Image restoration methods can be broadly classified as data‐based (DB) and model‐based (MB). DB approaches learn the restoration process directly from training data,^11^, ^12^, ^13^, ^14^ whereas MB approaches rely on mathematical descriptions of image degradation and prior knowledge of the imaging system.^15^, ^16^, ^17^, ^18^ DB methods often require large and representative training datasets, and their generalization performance may be affected when applied to data that differ substantially from those represented during training. This limitation may be partly mitigated by using sufficiently large and diverse datasets. In contrast, MB methods do not require training datasets, but their performance depends on the adequacy of the physical model and the accuracy of its parameter estimates. Consequently, model mismatch or inaccurate parameter estimation may compromise restoration performance.

In mammography, several MB restoration pipelines have demonstrated the potential to reduce image noise while preserving clinically relevant image structures.^16^, ^19^, ^20^, ^21^ However, these approaches have generally relied on noise models that primarily represent quantum and electronic noise components. Detector‐related structural noise and spatial noise correlation are not explicitly represented in these simplified models, although both may affect noise magnitude, noise texture, and restoration performance.^22^, ^23^, ^24^

Previous mammographic restoration studies have primarily evaluated performance using physical image‐quality metrics and model observers.^16^, ^19^, ^21^, ^25^, ^26^ More broadly, human‐observer studies have investigated the effects of radiation dose, noise, spatial resolution, and image processing on mammographic interpretation and lesion detection, including evaluations performed by breast radiologists.^27^, ^28^, ^29^, ^30^, ^31^ Other controlled detection studies have employed medical physicists as human observers.^15^, ^32^ Nevertheless, evidence regarding the effect of MB mammographic image restoration on lesion detectability specifically assessed by breast radiologists remains limited.

Microcalcification clusters (MCs) represent a clinically relevant detection task in breast cancer screening because these subtle findings must often be identified in low‐contrast and anatomically complex backgrounds. Their visibility and detectability are particularly sensitive to image noise and spatial resolution.^28^, ^31^ Therefore, MC detection provides a suitable task for assessing whether noise reduction through image restoration produces a measurable improvement in human‐observer performance.

In this study, we present an extended MB restoration pipeline that incorporates quantum, electronic, and structural noise components, as well as spatial noise correlation, into the denoising procedure. The pipeline was applied to clinical DM images acquired using a GE Senographe Pristina system and configured to target the signal and noise characteristics predicted by the model for acquisitions performed at approximately twice the original radiation dose. The objective was to determine whether the proposed restoration framework improves the detectability of simulated MCs relative to the original images. We hypothesized that restoration would increase detection performance under the controlled experimental conditions investigated. The effect of restoration was evaluated in a human‐observer study involving six breast radiologists using a four‐alternative forced‐choice (4AFC) protocol.

## 2 MATERIALS AND METHODS

This section describes the methodology adopted in this work. First, the proposed MB restoration pipeline and the noise model are presented. Next, we describe the human observer study used to evaluate the effect of image restoration on microcalcification detectability.

### 2.1 Model‐based image restoration pipeline

The model‐based image restoration pipeline proposed in this study extends a previously developed restoration framework.^19^ Originally formulated for a linear noise model comprising only quantum and electronic components, the framework was adapted here to a quadratic noise model commonly used to describe signal‐dependent noise variance in DM^33^, ^34^ and extended to explicitly account for spatial noise correlation.

The quadratic noise model accounts for the three main noise sources in mammography systems: quantum, electronic, and structural noise. Quantum noise follows Poisson statistics, whereas the electronic and structural components are modeled here as Gaussian random variables. At photon‐count levels typical of clinical mammography, the Poisson component can be well approximated by a Gaussian distribution according to the central limit theorem.^35^, ^36^ Thus, conditional on the underlying noise‐free signal, the aggregate noise at each pixel is modeled as a zero‐mean Gaussian random variable with signal‐dependent variance.^36^, ^37^

Beyond this variance model, noise in DM also exhibits spatial correlation, that is, the noise values at neighboring pixels are not statistically independent. This correlation arises from the image acquisition chain and is particularly pronounced in indirect‐conversion detectors because of lateral light spread within the scintillator.^22^

Accordingly, to consider the quadratic noise model as well as the spatial noise correlation, we model the value at pixel position i in a raw mammography image acquired at a dose level of x% (relative to the original clinical dose) as

(1) $$\begin{array}{cc} Z_{x\%}\left(i\right) & =y\left(i\right)+\sigma \left(i,y\right)\left(\eta *\kappa_{n}\right)\left(i\right)+\tau ,\;\eta \left(i\right)∼N\left(0,1\right),\\ & \;\kappa_{n}=\frac{\kappa }{{∥\kappa ∥}_{2}}, \end{array}$$

where $Z_{x\%}$ denotes the DM image; y is the noise‐free image, proportional to the energy of the X‐rays reaching the detector; τ is a constant offset added to prevent negative values in the digital image; η is a zero‐mean, unit‐variance white Gaussian random field resulting from the Gaussian approximation under clinical exposure conditions^35^, ^36^; and σ is a function of y that defines the local noise standard deviation. The symbol ∗ denotes the convolution operation, and the kernel $\kappa_{n}$ models the spatial correlation introduced by the detector and acquisition system. Note that the correlation kernel is normalized such that $∥\kappa_{n}{∥}_{2}=1$, ensuring that the noise variance is preserved after convolution. The expectation and variance of $Z_{x\%}$ are given by

(2) $$\begin{array}{cc} E\left\{Z_{x\%}\left(i\right)|y\left(i\right)\right\} & =y(i)+\tau ,\\ \;\mathrm{var}\left\{Z_{x\%}\left(i\right)|y\left(i\right)\right\} & =\xi_{s}^{2}y{\left(i\right)}^{2}+\xi_{q}\left(i\right)y\left(i\right)+\xi_{e}^{2}, \end{array}$$

where $\xi_{s}$ is the coefficient associated with the standard deviation of the structural noise component, attributed to detector non‐uniformities; $\xi_{q}$ corresponds to the quantum noise component, related to quantum efficiency and gain in image formation, and varies with the pixel position i; and $\xi_{e}$ represents the standard deviation of the electronic noise.

To model an image corresponding to a higher radiation dose at level βx%, we introduce a multiplicative factor β>1. Under this formulation, the tube current–time product (mAs) is increased by a factor β, while the tube voltage, target/filter combination, and other acquisition parameters remain fixed. Because the detector signal is proportional to the mAs under a fixed acquisition configuration, the noise‐free signal y is also scaled by β. The expectation and variance of the resulting modeled higher‐dose image, denoted $Z_{\beta x\%}$, are given by

(3) $$\begin{array}{cc} E\left\{Z_{\beta x\%}\left(i\right)|y\left(i\right)\right\} & =\beta y(i)+\tau ,\\ \;\mathrm{var}\left\{Z_{\beta x\%}\left(i\right)|y\left(i\right)\right\} & =\xi_{s}^{2}{\left(\beta y(i)\right)}^{2}+\xi_{q}\left(i\right)\left(\beta y(i)\right)+\xi_{e}^{2}, \end{array}$$

where βx% represents the modeled dose level obtained by scaling the original mAs by a factor β while maintaining the remaining acquisition parameters fixed.

Using the statistical models defined above for the original image $Z_{x\%}$ and the modeled higher‐dose image $Z_{\beta x\%}$, the previously developed MB restoration framework^19^ was extended to incorporate structural noise and spatial noise correlation. The extended framework was applied to the original image $Z_{x\%}$ to generate the restored image ${\hat{Z}}_{\beta x\%}$, designed to reproduce the signal and noise characteristics of the modeled higher‐dose image $Z_{\beta x\%}$. Figure 1 illustrates the restoration pipeline employed in this study.

**FIGURE 1:**
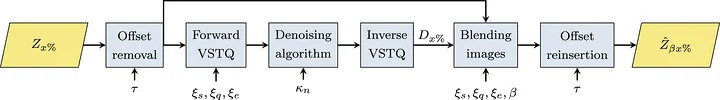
The main pipeline used in this work to restore the mammography images.

The restoration pipeline begins by subtracting the constant offset (τ) from the original image $Z_{x\%}$. Because the noise model used to describe both the original and modeled higher‐dose images is signal‐dependent, we employed a variance‐stabilizing transformation for quadratic noise models (VSTQ).^36^ This transformation aims to convert signal‐dependent noise into approximately signal‐independent noise. The VSTQ applied to $Z_{x\%}$, after the subtraction of the offset, is defined as^36^

(4) $$F\left(Z_{x\%}\right)=\frac{\ln\left(\left|2\xi_{s}\sqrt{\xi_{s}^{2}{\left(Z_{x\%}\left(i\right)-\tau \right)}^{2}+\xi_{q}\left(i\right)\left(Z_{x\%}\left(i\right)-\tau \right)+\xi_{e}^{2}}+2\xi_{s}^{2}\left(Z_{x\%}\left(i\right)-\tau \right)+\xi_{q}\left(i\right)\right|\right)}{\xi_{s}},$$

where $F(Z_{x\%})$ denotes the stabilized image.^1^

After stabilization, the restoration pipeline proceeds to the denoising step. At this stage, the noise of the stabilized image can be treated as approximately an additive Gaussian noise with unit variance, allowing the use of denoising algorithms originally designed for Gaussian noise. However, to be consistent with the adopted noise model, the denoising algorithm must explicitly account for the spatial correlation. In this work, we selected the Block‐Matching and 3D Filtering (BM3D) algorithm adapted for correlated noise.^38^ This method uses the noise power spectrum associated with the correlation kernel to compute the transform‐domain noise variances for each group of similar patches, thereby adapting shrinkage and patch matching to spatially correlated noise.

Once the filtering step is complete in the stabilized domain, the pipeline must return the image to the initial intensity scale. This is achieved by applying the inverse of VSTQ to obtain the denoised image $D_{x\%}$. The goal is to determine a mapping $F^{-1}$ such that

(5) $$F^{-1}:E\left\{F(Z_{x\%})|y\right\}\to E\left\{Z_{x\%}-\tau |y\right\}=y.$$

Since a closed‐form expression for the inverse mapping defined in (Eq. 5) is not available, it was computed numerically via Monte Carlo simulation.^36^ The procedure to estimate $F^{-1}$ is based on multiple noisy realizations of a signal generated according to the quadratic noise model, followed by application of the VSTQ. Then, the expected values of both the stabilized signal $E\left\{F(Z_{x\%})|y\right\}$ and the offset‐corrected noisy signal in the initial domain $E\{Z_{x\%}-\tau |y\}$ are estimated through Monte Carlo averaging. These estimates are used to construct a look‐up table (LUT), where the inputs consist of the stabilized signal $F(Z_{x\%})$ and the coefficient $\xi_{q}$, while the output corresponds to the ground‐truth signal y. Intermediate values not present in the table are computed via first‐order 2D interpolation.

Once the denoised image in the VSTQ domain is transformed back to the initial intensity domain via $F^{-1}$, yielding $D_{x\%}$, the restoration pipeline proceeds to its final stage: a blending step that combines the original ($Z_{x\%}$) and denoised images ($D_{x\%}$). This process uses a weighted combination to produce the restored image ${\hat{Z}}_{\beta x\%}$, designed to reproduce the signal and noise characteristics of the modeled higher‐dose image $Z_{\beta x\%}$. The blending step is defined as

(6) $${\hat{Z}}_{\beta x\%}\left(i\right)=\rho \left(i\right)\left(Z_{x\%}\left(i\right)-\tau \right)+\bar{\rho }\left(i\right)D_{x\%}\left(i\right)+\tau ,$$

where ρ and $\bar{\rho }$ are spatially varying blending weights, $D_{x\%}$ is the denoised version of the input image, and ${\hat{Z}}_{\beta x\%}$ represents the final restored image after blending, with the offset τ reinserted after blending. The estimation of the blending weights is based on the ratio between the variances of the modeled higher‐dose image $Z_{\beta x\%}$ (Eq. 3) and the original image $Z_{x\%}$ (Eq. 2), and is given by

(7) $$\begin{array}{cc} \rho (i) & =\sqrt{\frac{\mathrm{var}\left\{Z_{\beta x\%}\left(i\right)|y\left(i\right)\right\}}{\mathrm{var}\left\{Z_{x\%}\left(i\right)|y\left(i\right)\right\}}}\\ & =\sqrt{\frac{\xi_{s}^{2}{\left(\beta y(i)\right)}^{2}+\xi_{q}\left(i\right)\left(\beta y(i)\right)+\xi_{e}^{2}}{\xi_{s}^{2}y{\left(i\right)}^{2}+\xi_{q}\left(i\right)y\left(i\right)+\xi_{e}^{2}}},\;\bar{\rho }\left(i\right)=\beta -\rho \left(i\right). \end{array}$$

It is important to note that the true noise‐free image y is not available in the clinical environment. Therefore, we approximate it by using the denoised version, such that $y\approx D_{x\%}$, to compute the weights, resulting in the restored image ${\hat{Z}}_{\beta x\%}$. This MB restoration pipeline, adapted for the quadratic noise model as well as for spatial correlation, was used to evaluate its effect on microcalcification detectability in the human observer study.

### 2.2 Human observer study

The human observer study consisted of four sequential stages: dataset preparation, pilot study, main observer study, and statistical analysis. Dataset preparation involved selecting clinical mammograms, simulating and inserting microcalcification clusters at multiple contrast levels, and organizing the images for the observer experiments. The pilot study was conducted to estimate a reader‐specific contrast threshold for each radiologist. These thresholds were subsequently used to generate reader‐specific datasets for the main study, with the aim of achieving approximately comparable baseline task difficulty across readers. During the main study, each radiologist independently evaluated both the original and restored image conditions. Finally, the reader responses were analyzed to assess the effect of image restoration on microcalcification detectability. Each stage of the experimental workflow is described in detail below.

A total of six breast radiologists participated in the human observer studies evaluating the proposed image restoration pipeline. Their experience in breast imaging ranged from 1 to 25 years. For descriptive reporting and a complementary assessment of estimated individual contrast thresholds during the pilot study, the readers were classified as Junior (early‐career; 1 year of experience) or Senior (experienced; 12–25 years of experience), as summarized in Table 1. The readers were from two clinical institutions involved in the study: Skåne University Hospital (Sweden) and Barretos Cancer Hospital (Brazil). Each radiologist independently evaluated both the original and restored images in the main observer study.

**TABLE 1:** Radiologist experience and participants' institutional affiliation.

	Junior			Senior		
Radiologist	1*	2*	3*	4*	5**	6**
**Experience (years)**	1	1	1	12	25	19

* Radiologists from Skåne University Hospital (Sweden).

** Radiologists from Barretos Cancer Hospital (Brazil).

#### 2.2.1 Image dataset preparation

Figure 2 summarizes the image dataset preparation workflow and the subsequent pilot study. Starting from the raw clinical DM images, simulated microcalcification clusters were inserted at multiple contrast levels, followed by ROI extraction and dataset organization. The resulting original image ROI datasets were then used in the 4AFC pilot study with the 3D1U staircase procedure to estimate the contrast threshold for each radiologist.

**FIGURE 2:**
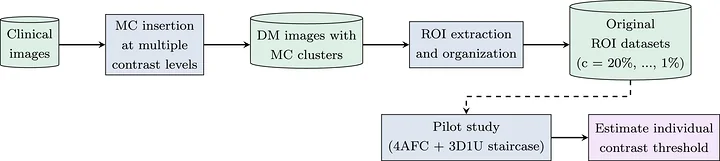
Overview of the workflow for image dataset preparation and reader‐specific contrast‐threshold estimation during the pilot study.

A total of 120 unprocessed (raw) clinical DM images from examinations assigned BI‐RADS assessment category 1 (negative) or 2 (benign) and breasts classified as BI‐RADS density category C (heterogeneously dense) or D (extremely dense) were provided by Barretos Cancer Hospital (Brazil). The retrospective study was approved by the institutional Research Ethics Committee (CAAE #78625417.1.1001.5437). All images were anonymized before analysis, and the requirement for informed consent was waived because of the retrospective nature of the study and the use of previously acquired anonymized images.

Examinations assigned BI‐RADS assessment categories 1 or 2 were selected to exclude suspicious findings from the source examinations, whereas breasts assigned density categories C or D were selected to provide more challenging anatomical backgrounds for the microcalcification detection task. The images were acquired under routine clinical conditions using a GE Senographe Pristina mammography system. This system employs an amorphous silicon (a‐Si) detector coupled to a CsI:Tl scintillator, in which lateral light spread contributes to spatial noise correlation.^24^, ^39^, ^40^

All images were acquired using the Automatic Optimization of Parameters (AOP) mode. Examination of the DICOM header information showed that all images included in the study were acquired using a rhodium target/silver filter combination (Rh/Ag) at 34 kVp. The mean tube current–time product was 49.9 ± 17.0 mAs (range: 24.3–113.2 mAs), and the mean compressed breast thickness was 6.2 ± 1.0 cm (range: 3.4–8.5 cm).

The simulation and insertion of MCs in this study were performed using previously developed in‐house software.^15^, ^41^, ^42^ The insertion framework has been previously validated in a 2‐AFC human observer study. A total of 50 individual microcalcifications extracted from clinical mammograms acquired on the GE Senographe Pristina system were used to generate the simulated clusters. Individual microcalcifications were manually segmented by a medical physicist from regions containing isolated calcifications. The pixels inside the segmented area were extracted to create lesion templates, whereas the surrounding background pixels were set to zero. The template intensities were then normalized by their maximum pixel value, preserving the lesion morphology and relative intensity distribution while standardizing the template amplitude for the insertion procedure. The resulting microcalcification templates had diameters ranging from approximately 140 to 350 μm.

Clusters were generated within 100 × 100 pixel windows (10.0 mm × 10.0 mm). Within each window, a bivariate Gaussian probability distribution was defined, with its mean at the window center and a standard deviation of 60 pixels (6.0 mm). This probability distribution was used to determine the spatial positions of the six microcalcifications selected from the set of 50 clinical templates. To avoid overlap and excessive proximity between microcalcifications, after placing each template, a 40 × 40 pixel inverted bivariate Gaussian mask (4.0 mm × 4.0 mm), with a standard deviation of 5 pixels, was applied to the probability distribution at the selected location. This iterative procedure reduced the probability of placing subsequent microcalcifications near previously selected positions, resulting in spatially distributed cluster configurations. Figure 3 illustrates the updated probability distribution after each successive placement and the final simulated cluster mask.

**FIGURE 3:**
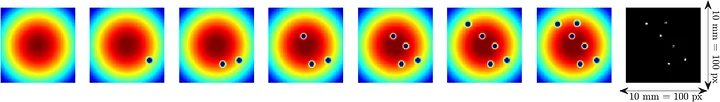
Sequential probability maps used to determine the positions of six microcalcifications within the cluster window and the resulting cluster mask.

A total of 100 clusters were generated. Of the 120 available clinical images, 100 were randomly selected for lesion insertion, and one simulated cluster was inserted into each selected image. Insertion locations near the skin, chest wall, and pectoral muscle were excluded. Possible insertion locations were identified using the dense‐tissue masks generated by the Laboratory for Individualized Breast Radiodensity Assessment (LIBRA) software, which provides a binary mask indicating the presence of dense tissue at each spatial location in the mammogram.^43^ Although all mammograms were classified globally as BI‐RADS density category C or D, the LIBRA masks were used to restrict cluster insertion to locally dense fibroglandular regions. Thus, the combination of BI‐RADS density classification and LIBRA local tissue segmentation was used to create a challenging detection task in dense anatomical backgrounds. Because BI‐RADS 2 examinations may contain benign findings, the candidate insertion regions in the original images were reviewed by a breast radiologist before lesion insertion. Regions containing pre‐existing lesions, recognizable benign findings, or ambiguous anatomical structures that could interfere with the detection task were excluded. This procedure ensured that the simulated microcalcification clusters constituted the only lesion targets within the selected regions.

The insertion process was performed by increasing the attenuation in the mammography image at the pixels corresponding to the lesion locations. This was achieved through multiplication of the original pixel values by a factor derived from the cluster mask^15^, ^41^

(8) $$Z_{x\%}\left(i\right)=Z_{x\%}^{o}\left(i\right)\left(1-cS(i)\right),$$

where $Z_{x\%}^{o}$ is the raw DM image acquired at the original dose without simulated clusters; $Z_{x\%}$ is the resulting image after lesion insertion; i denotes the pixel position ; c is the lesion contrast; and S is the cluster mask.

To generate different cluster contrast levels, the parameter c ranged from 20% to 1%, decreasing by 15% between successive levels. For each contrast level, a separate image database was generated. Each database contained the same 100 clinical DM images, each with its corresponding simulated microcalcification cluster inserted at an identical spatial location. Thus, the only difference between databases was the contrast of the inserted clusters.

A key advantage of this insertion method is that it provides precise control over the contrast (c) of each simulated cluster while preserving the anatomical background of the clinical mammogram. Higher contrast values increase cluster visibility, whereas lower values produce progressively more challenging detection tasks. This flexibility was particularly important for the pilot study because each cluster pattern could be generated at multiple contrast levels, enabling the estimation of a reader‐specific contrast threshold for each radiologist. The contrast‐threshold estimation procedure is described in Section 2.2.2. Figure 4 shows the same mammographic region with the simulated cluster inserted at different contrast levels.

**FIGURE 4:**
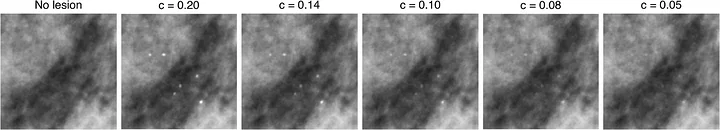
From left to right, the same mammographic region shown without an inserted lesion (0%) and with a simulated microcalcification cluster at contrast levels of 20%, 14%, 10%, 8%, and 5%.

To ensure proper image interpretation by the radiologists, all images were processed using the same GE Senographe Pristina system at Barretos Cancer Hospital, where they had originally been acquired, converting the “for processing” images into the corresponding “for presentation” format. After conversion to the “for presentation” format, one 300 × 300 pixel lesion‐containing region of interest (ROI) was extracted from each of the 100 images selected for cluster insertion, yielding 100 lesion‐containing ROIs. The same regions were extracted from the corresponding original and restored images, and the clusters were not necessarily centered within the ROIs. This procedure was repeated for all contrast levels evaluated in the study.

In addition, 300 background ROIs without simulated microcalcifications were extracted from locally dense breast regions identified by LIBRA. To increase anatomical background variability, these ROIs were sampled across all 120 available clinical images, excluding regions used for cluster insertion. The background ROIs were selected using the same criteria as the lesion‐containing ROIs, except that no simulated clusters were present. They were also reviewed by a breast radiologist, and regions containing suspicious findings, recognizable benign findings, or ambiguous anatomical structures were excluded. The extracted cluster and background ROIs were subsequently organized into datasets for the pilot observer study. For each contrast level, a dedicated dataset containing 400 ROIs was generated: 100 lesion‐containing ROIs and 300 background ROIs. Within each contrast‐level dataset, the 300 background ROIs were randomly assigned without replacement to the 100 lesion‐containing ROIs, yielding 100 4AFC cases, each comprising one lesion‐containing ROI and three background ROIs.

An in‐house MATLAB graphical user interface (GUI) was used to conduct both the pilot and main observer studies. The GUI displayed the ROIs simultaneously in a 4AFC layout, randomized their positions across trials, recorded the readers' responses, and provided immediate feedback after each selection. All observer sessions were performed in clinical environments using high‐resolution workstations routinely employed for mammographic interpretation. Ambient lighting conditions followed the clinical protocols of each institution. Radiologists were free to choose their viewing distance, whereas zooming and window/level adjustments were disabled to standardize the evaluation conditions. No time limit was imposed for image interpretation. After each response, the software automatically recorded the selected ROI.

#### 2.2.2 Pilot study

The database containing multiple contrast levels was used in the pilot study to estimate a reader‐specific contrast threshold for each radiologist, as illustrated in Figure 2.

The goal of the pilot study was to estimate a reader‐specific contrast threshold that would set each radiologist's performance within a prespecified target range of approximately 75%–80% correct responses when detecting microcalcification clusters in the original image dataset. This target performance range is commonly adopted in psychophysical experiments using forced‐choice paradigms because it provides stable performance while remaining sensitive to changes in task difficulty.^44^, ^45^ Performance within this range also reduces floor and ceiling effects, thereby maximizing sensitivity to potential differences between imaging conditions.^32^ The resulting reader‐specific contrast threshold was subsequently used to define the cluster insertion contrast in the main observer study, with the aim of achieving approximately comparable baseline task difficulty across readers.

The conventional three‐down‐one‐up (3D1U) staircase theoretically converges to approximately 79% correct responses on the psychometric function.^45^ Because this convergence level lies within the prespecified target range of 75%–80%, the 3D1U procedure was selected for the adaptive estimation of the reader‐specific contrast thresholds and combined with the 4AFC paradigm.^46^, ^47^ In each trial, observers were presented simultaneously with four ROIs: one containing a microcalcification cluster and three without lesions. The task was to identify the ROI containing the cluster.

The microcalcification cluster contrast was adaptively adjusted according to the 3D1U rule. After three consecutive correct responses, the contrast was decreased by one step for the subsequent trial, corresponding to a 15% reduction relative to the preceding contrast level. After an incorrect response, the contrast was increased by one step by returning to the preceding higher‐contrast level. To reduce the influence of the initial adaptation period, the reader‐specific contrast threshold was estimated as the mean of the contrast levels presented during the final 60 trials, after discarding the first 40 trials. The image datasets containing the different cluster contrast levels generated during dataset preparation (Section 2.2.1) were used to support this adaptive procedure.

Each radiologist completed a pilot session consisting of 100 4AFC cases, with four ROIs presented per case, using only original images. No separate training session was performed; instead, the pilot study itself served to familiarize the readers with the 4AFC task.

#### 2.2.3 Main observer study

Figure 5 summarizes the workflow adopted to generate individual datasets with original and restored images for each reader in the main observer study.

**FIGURE 5:**
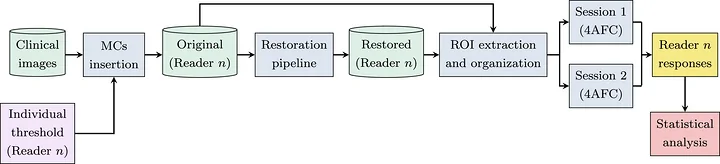
Overview of the workflow used to generate reader‐specific original and restored image datasets in the main study. The resulting responses were used for statistical analysis.

For the main observer study, new image databases were generated. For each radiologist, the reader‐specific contrast threshold estimated during the pilot staircase procedure was used to define the insertion contrast of the simulated microcalcification clusters, thereby tailoring task difficulty to the reader's detection capability. To minimize memory effects, the clusters were inserted into new locations, guided by the binary mask of dense regions estimated from LIBRA, which had not been used during the pilot study. The same 100 clinical images and cluster locations were used across all readers. Only the inserted cluster contrast differed among the reader‐specific datasets according to the threshold estimated for each radiologist. The resulting images with inserted MC clusters constituted the original image datasets.

The proposed MB restoration pipeline (Section 2.1) was applied to all original image datasets, generating the corresponding restored image datasets. The noise‐model parameters required by the restoration framework ($\xi_{s}$, $\xi_{q}$, $\xi_{e}$, κ, and τ) were estimated from uniform phantom images acquired at multiple exposure levels using the same physical GE Senographe Pristina unit, Rh/Ag target/filter combination, and tube voltage of 34 kVp used for the clinical images, following the protocols described in^22^, ^36^.

Because the inverse mapping defined in (Eq. 5) does not have a closed‐form expression, the procedure described in Section 2.1 was used to construct a numerical LUT. Specifically, $10^{5}$ equally spaced values of y were sampled over the range $\left[0,\;1.65\times 10^{4}\right]$, corresponding to the dynamic range of the mammography images in our dataset. In addition, $10^{3}$ equally spaced values of $\xi_{q}$ were sampled over the interval [0.0391,0.1148], consistent with the range estimated across the detector from the uniform phantom measurements. For each $\left(y,\xi_{q}\right)$ pair, $10^{5}$ realizations of the degraded signal $Z_{x\%}$ were generated using the quadratic noise model (Eq. 2). The VSTQ (Eq. 4) was then applied to all realizations, and the expected values $E\{F\left(Z_{x\%}\right)|y\}$ and $E\{Z_{x\%}-\tau |y\}$ were estimated. These values formed the LUT used to approximate the inverse transformation numerically.

In this study, the restoration pipeline was configured with a dose multiplication factor of β=2, generating restored images ${\hat{Z}}_{200\%}$ from the original clinical mammograms $Z_{100\%}$. Consequently, the restored images were configured to target the signal and noise characteristics predicted for acquisitions at twice the original radiation dose. The selection of β=2 was based on previous work,^16^ in which this dose multiplication factor demonstrated improved detectability in a model‐observer study.

After restoration, both the original and restored “for processing” images were converted into the corresponding “for presentation” format using the same GE Senographe Pristina system employed for the original clinical acquisitions. The original DICOM metadata and acquisition parameters were preserved throughout this procedure, and the manufacturer's proprietary image processing was applied identically to both image sets. The same ROI extraction and dataset organization procedures adopted in the pilot study were then applied to both image conditions. This resulted in one paired reader‐specific dataset comprising two image conditions (original and restored), each containing 400 ROIs.

The main study followed the same 4AFC paradigm, with each case comprising one ROI containing a microcalcification cluster and three background ROIs. The task was to identify the ROI containing the cluster. Each radiologist completed two reading sessions separated by at least five days, with 100 cases presented per session. For each reader independently, 50 of the 100 cases were randomly assigned to the original image condition ($Z_{100\%}$) and the remaining 50 to the restored image condition (${\hat{Z}}_{200\%}$) in the first session. In the second session, each case was presented under the complementary image condition. Thus, across the two sessions, each case was evaluated exactly once under each image condition by the same reader. The presentation order was randomized independently for each reader within each session, and the radiologists remained blinded to the image condition.

The percentage of correct responses (PC) was calculated separately for each radiologist and image condition as the number of correctly identified cases divided by the total number of cases evaluated under that condition, multiplied by 100%. The mean PC across readers was then calculated for each image condition for descriptive comparison.

#### 2.2.4 Statistical analysis

Because each radiologist evaluated multiple image cases, the observer study generated repeated binary outcomes (correct or incorrect responses). Therefore, a generalized linear mixed model (GLMM) was used as the primary inferential analysis.^48^, ^49^ The model was fitted using the fitglme function in MATLAB R2025b. It assumed a binomial distribution with a logit link function and included image condition (original or restored) as a fixed effect, while reader and image case were modeled as crossed random intercepts. Original images were used as the baseline category for image condition. Model parameters were estimated by maximum pseudo‐likelihood, and the effect of image restoration was summarized using odds ratios (ORs) with corresponding 95% confidence intervals.

A second GLMM was fitted using only the original image condition to evaluate the effect of reader experience on baseline observer performance. Junior radiologists were used as the reference category. A significance level of 0.05 was adopted for all statistical analyses.

## 3 RESULTS

### 3.1 Estimated noise parameters and restoration examples

The noise parameters estimated in this work are: $\xi_{s}=0.0015$, $\xi_{e}=2.6$, and τ=0.5. Figure 6 shows the quantum gain map $\xi_{q}$ and the normalized correlation kernel $\kappa_{n}$. Figure 7 shows an example of a selected ROI acquired at the original dose $Z_{100\%}$, the denoised image $D_{100\%}$, and the final restored image ${\hat{Z}}_{200\%}$. The upper row shows the visual appearance of the three versions, with a square indicating the selected region. The lower row shows magnified views of the highlighted regions.

**FIGURE 6:**
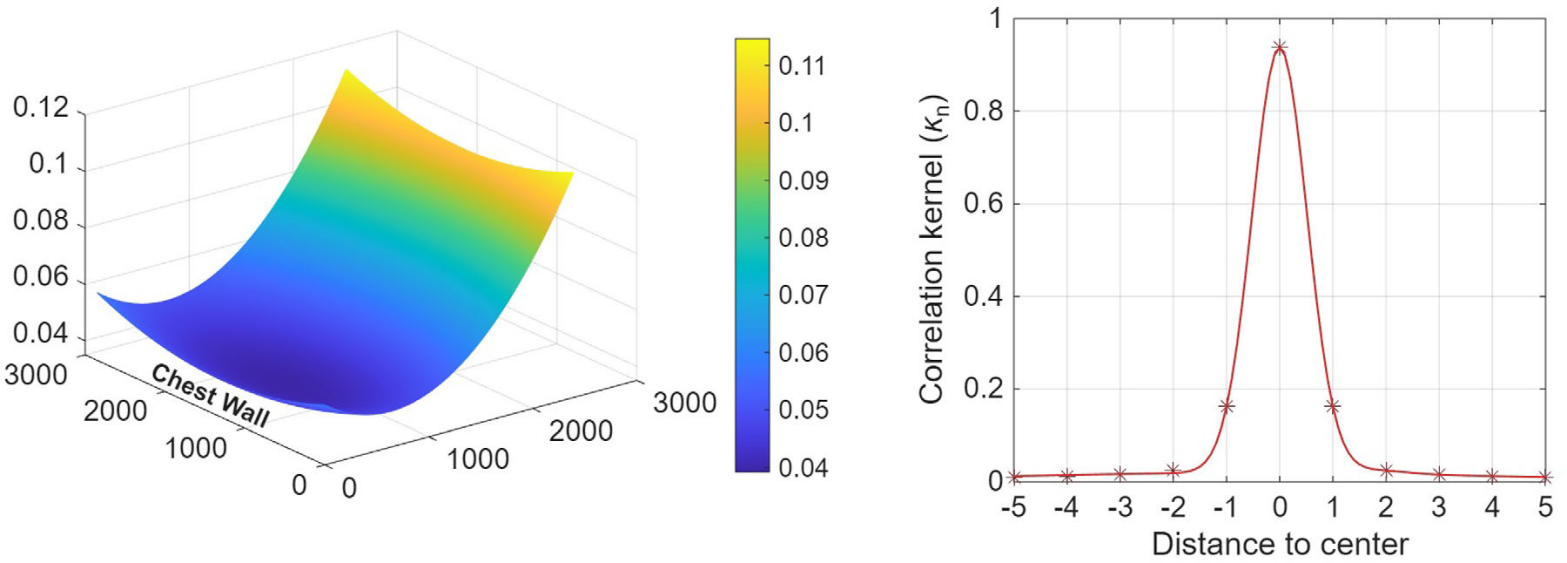
Estimated noise parameters: spatially dependent quantum gain $\xi_{q}$ (left), and normalized convolution kernel $\kappa_{n}$ (right).

**FIGURE 7:**
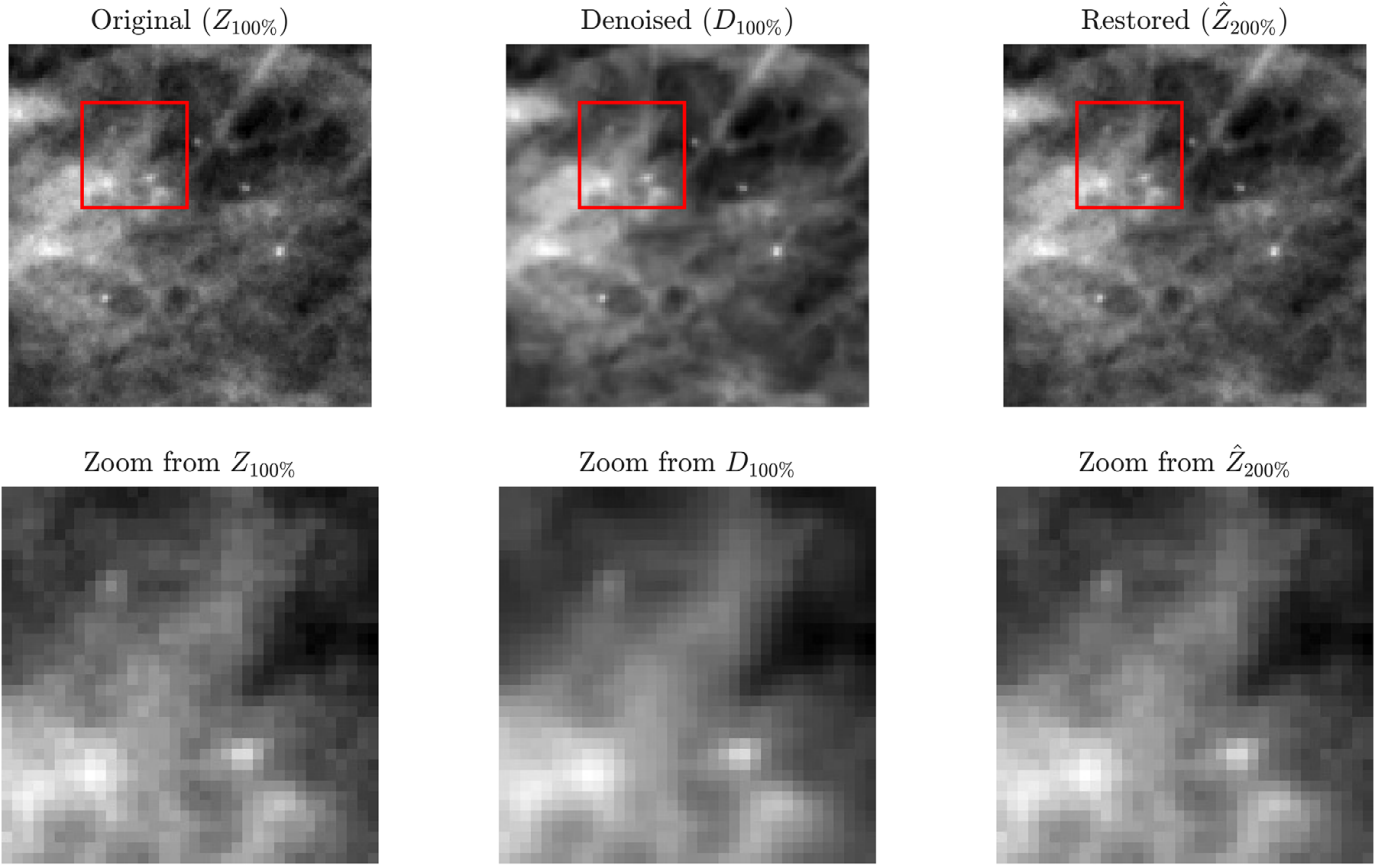
Example of a mammographic ROI containing a simulated microcalcification cluster. From left to right: original, denoised, and restored images. The lower row shows magnified views of the regions indicated by the red squares in the upper row.

### 3.2 Human observer study

Figure 8 shows the progression of contrast values across trials for each reader. The mean contrast level (c) across the final 60 trials was used to estimate each reader's contrast threshold. The light‐blue points represent the initial 40 trials, which were excluded from the calculation, whereas the dark‐blue highlighted points represent the final 60 trials used to estimate the contrast threshold reported in each panel. At the beginning of the staircase procedure, the relatively high initial contrast made the detection task easier, resulting in a rapid decrease in contrast. As the contrast decreased and task difficulty increased, the staircase values began to fluctuate around each reader's contrast threshold, which was intended to yield performance within the prespecified target range of approximately 75%–80% correct responses.

**FIGURE 8:**
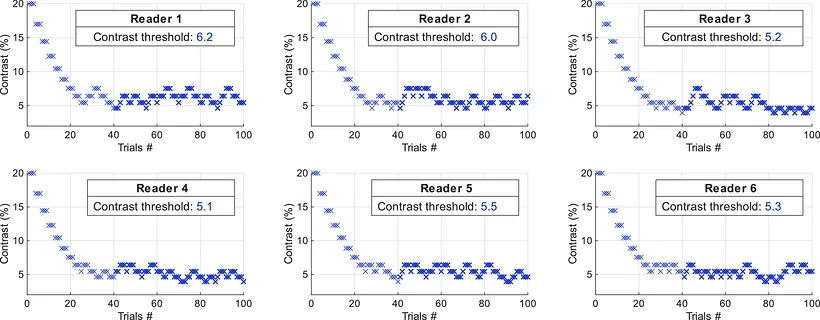
Contrast threshold estimation for each radiologist during the pilot study. The light‐blue points represent the initial 40 trials, which were excluded from the calculation, whereas the dark‐blue highlighted points represent the final 60 trials used to estimate the reader‐specific contrast threshold.

Table 2 presents the individual contrast thresholds estimated for each radiologist during the pilot study using the original image database. The 95% confidence intervals (CIs) for the group and overall mean thresholds were calculated using Student's t distribution based on the between‐reader variability. Junior radiologists had a mean threshold of 5.8% (95% CI: 4.5–7.1%), while senior radiologists had a mean threshold of 5.3% (95% CI: 4.8–5.8%). Across all six radiologists, the mean contrast threshold was 5.6% (95% CI: 5.1–6.0%).

**TABLE 2:** Reader‐specific microcalcification contrast thresholds (%) estimated during the pilot study using the original image database. These thresholds were used to define the cluster insertion contrast for each radiologist in the main observer study. The 95% confidence intervals for the group and overall mean thresholds were calculated using Student's t distribution based on between‐reader variability.

	Contrast threshold per radiologist (%)						Mean [95% CI]		
Image condition	Junior			Senior			Junior	Senior	Overall
	1	2	3	4	5	6			
Original	6.2	6.0	5.2	5.1	5.5	5.3	5.8 [4.5, 7.1]	5.3 [4.8, 5.8]	5.6 [5.1, 6.0]

Table 3 summarizes the PC obtained by each radiologist under the original and restored image conditions. Considering the six readers, the mean PC increased from 78.3% for the original images to 84.3% for the restored images, corresponding to an absolute improvement of 6.0 percentage points. Five radiologists showed higher PC values after restoration, whereas one radiologist achieved the same PC under both conditions. The main study datasets were generated using the reader‐specific contrast thresholds estimated during the pilot study, which aimed to place reader performance within the prespecified target range of 75%–80% correct responses under the original‐image condition. The overall mean PC of 78.3% for the original images fell within this range, supporting the adequacy of the reader‐specific calibration.

**TABLE 3:** Percentage of correct responses (PC) for the original and restored image conditions in the main observer study. Radiologists were grouped by experience level as Junior (1 year of experience) or Senior (12 years or more). The 95% confidence intervals (CIs) for the group and overall mean PC values were calculated using Student's t distribution based on between‐reader variability.

	PC per radiologist (%)						Mean [95% CI]		
Image condition	Junior			Senior			Junior	Senior	Overall
	1	2	3	4	5	6			
Original	81	79	76	75	73	86	78.7 [72.4, 84.9]	78.0 [60.6, 95.4]	78.3 [73.4, 83.3]
Restored	87	85	86	82	80	86	86.0 [83.5, 88.5]	82.7 [75.1, 90.3]	84.3 [81.5, 87.2]
PC improvement (percentage points)	6	6	10	7	7	0	7.3	4.7	6.0

Figure 9 presents the responses of all radiologists across the 100 evaluated cases in the main study. Panels (a) and (b) show the responses obtained using the original and restored images, respectively, while panel (c) presents the difference map between both conditions. In (a) and (b), blue cells indicate correct responses and red cells indicate incorrect responses. In panel (c), green cells represent responses that changed from incorrect to correct after restoration, orange cells indicate responses that changed from correct to incorrect, and white cells correspond to unchanged responses. Overall, the restoration yielded 506 correct responses, compared with 470 for the original images. Across the 600 reader‐case pairs, corresponding to 1,200 individual responses across the two image conditions, restoration changed 91 responses from incorrect to correct and 55 from correct to incorrect, while 454 responses remained unchanged, resulting in a gain of 36 correct detections.

**FIGURE 9:**
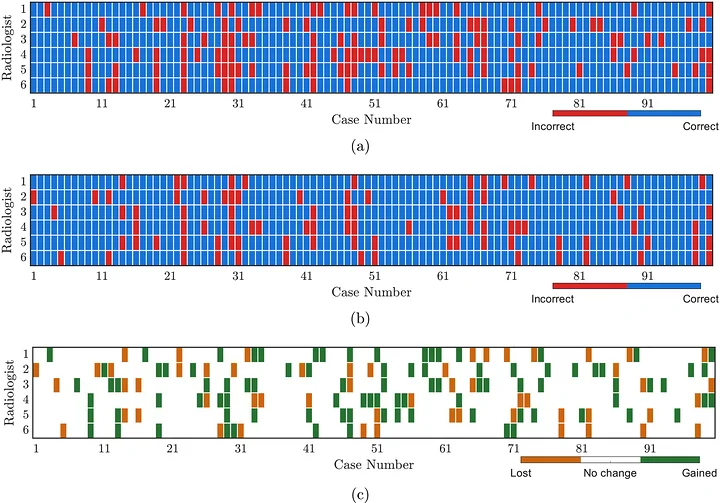
Responses of the six radiologists across the 100 cases in the main observer study: (a) original images, (b) restored images, and (c) changes in responses between the restored and original conditions.

Table 4 summarizes the statistical analyses performed in this study and their principal findings. The GLMM analysis indicated a significant effect of restoration on detection performance (OR = 1.57, 95% CI: 1.15–2.14, p=0.005), suggesting that restored images increased the odds of correct microcalcification detection by approximately 57% compared with the original images. A second GLMM was fitted to evaluate the effect of reader experience under the original image condition. The separate GLMM found no evidence of an experience‐group effect under the original‐image condition (p=0.858).

**TABLE 4:** Summary of the generalized linear mixed‐model analyses performed in this study.

Analysis	Comparison and effect estimate	Model	p‐value
Restoration effect	Restored versus original; OR = 1.57 (95% CI: 1.15–2.14)	GLMM	0.005
Reader experience (original images)	Senior versus Junior; OR = 0.96 (95% CI: 0.60–1.53)	GLMM	0.858

## 4 DISCUSSION

The present study evaluated an extended MB restoration framework incorporating quantum, electronic, and structural noise components, as well as spatial noise correlation. Its effectiveness was assessed through a human observer study. The primary GLMM showed a significant restoration effect (OR = 1.57, p=0.005), indicating that the proposed framework improved the detectability of simulated microcalcification clusters under the experimental conditions investigated.

The observed improvement is consistent with the physical foundation underlying the restoration framework. By accounting for the principal noise sources in DM and their spatial correlation, and by combining variance stabilization, correlated‐noise denoising, and image blending, the restoration process is designed to reduce noise while retaining lesion‐related signal components and anatomical information. This is particularly relevant for subtle microcalcification clusters whose detectability is strongly affected by image noise. However, because the present study did not isolate the effects of the individual processing components, the improvement cannot be attributed specifically to structural‐noise modeling, spatial‐correlation modeling, or any other individual component of the pipeline.

The present findings are consistent with previous investigations demonstrating the potential of MB restoration to improve mammographic image quality and lesion detectability. Early investigations primarily assessed restoration performance using physical image‐quality metrics and model observers. Brandão et al. (2025) reported improved detectability of simulated microcalcification clusters using a Channelized Hotelling Observer (CHO) after applying an MB restoration framework configured to emulate higher‐dose acquisitions. Similar findings were reported by Mota et al. (2023) using digital breast tomosynthesis (DBT) projection images, supporting the potential of MB restoration across different breast‐imaging modalities. In addition, Borges et al. (2020) demonstrated improved microcalcification detectability in a human observer study. However, the restoration framework evaluated in that study relied on a simplified noise model considering only quantum and electronic noise components. In contrast, the framework investigated here also incorporates structural noise and spatial noise correlation, thereby extending the physical description of image degradation. Although the present study was not designed to quantify the individual contribution of each additional modeling component, the extended framework was associated with improved microcalcification detectability under the experimental conditions investigated.

Although the present study focused on an MB restoration framework, these findings are also relevant in the broader context of mammographic image restoration. Deep learning methods have demonstrated strong performance in image restoration tasks.^12^, ^50^, ^51^, ^52^ However, their application in mammography remains challenging because realistic paired clinical datasets acquired at different radiation dose levels are difficult to obtain, as acquiring such data may require additional patient exposure. To address this limitation, data‐driven approaches commonly employ simulated training data, transfer learning, domain adaptation, or self‐supervised training strategies. Conversely, MB methods rely on physically motivated descriptions of the image degradation process and do not require large training datasets. Their performance, however, depends on the adequacy of the physical model and the accuracy of the estimated noise parameters. Therefore, the complementary strengths and limitations of model‐based and data‐driven approaches should be considered when developing image restoration strategies for mammography.

The interpretation of the findings is supported by the observer‐study design. The reader‐specific contrast thresholds estimated during the pilot study allowed the main study to be conducted at approximately comparable baseline detectability levels by adapting task difficulty to each reader's detection capability. The mean percentage of correct responses for the original images was 78.7% for Junior readers and 78.0% for Senior readers, with an overall mean of 78.3%. This value was within the prespecified target range of 75%–80% and close to the theoretical convergence level of approximately 79% for the 3D1U procedure. The separate GLMM restricted to the original‐image condition found no evidence of an experience‐group effect (p=0.858). The descriptive similarity between the groups was consistent with the intended reader‐specific calibration. Nevertheless, this result should not be interpreted as demonstrating statistical equivalence between Junior and Senior readers.

From a clinical perspective, the reported observer‐performance metrics should be interpreted within the context of this experimental design because the main observer study was performed using the reader‐specific contrast thresholds estimated during the pilot study. Specifically, the percentage of correct responses reflects detectability at these individualized contrast thresholds, corresponding to a predefined region of the psychometric function, rather than overall diagnostic performance across a range of decision thresholds. In addition, the 4AFC paradigm reduces the demands associated with lesion search and diagnostic decision‐making compared with routine interpretation of whole mammograms, although anatomical background structures can still affect lesion visibility. Against this controlled baseline, restoration produced an absolute increase of 6.0 percentage points in mean PC, representing a measurable improvement in lesion detectability under challenging imaging conditions. However, the magnitude of this benefit should not be interpreted as a direct improvement in clinical diagnostic accuracy. Further studies using ROC‐based observer paradigms, whole mammograms, and realistic clinical reading conditions are required to determine the extent to which these findings translate into improved diagnostic performance.

Despite the promising results, some limitations should be considered. The limited number of readers restricts the generalizability of the human observer findings and precludes a reliable assessment of whether the restoration effect differs according to reader experience. The experience‐group analysis was restricted to baseline performance in the original image condition and was not designed as an equivalence analysis. Therefore, its nonsignificant result neither establishes equivalent performance between Junior and Senior radiologists nor determines whether the restoration benefit is influenced by reader experience. Moreover, reader experience and institution were not fully balanced: all Junior readers were recruited from Skåne University Hospital, whereas the Senior group included one reader from Skåne and two from Barretos Cancer Hospital. Therefore, experience may be partly confounded with institutional or practice‐related factors.

Although consecutive reading sessions were separated by at least five days to reduce recall bias, some residual memory effects cannot be completely excluded. This interval is shorter than the longer washout periods used in previous clinical observer studies.^53^, ^54^ However, the use of small ROIs presented within a 4AFC paradigm, without broader anatomical context or complete mammographic images, likely reduced the memorability of individual cases and mitigated potential recall effects.

Immediate feedback was provided after each trial and may have promoted perceptual learning or adjustment of reading strategies during the sessions. Nevertheless, all readers were evaluated using the same protocol, the original and restored conditions were interleaved within each session, ROI positions were randomized across trials, and readers remained blinded to the image condition. These design features make it less likely that feedback systematically favored either condition. However, an influence of feedback on overall observer performance cannot be completely excluded.

Another limitation concerns the simulated lesions. The microcalcification templates were extracted from clinical mammograms and therefore represent the imaged appearance of microcalcifications rather than their true object morphology. Consequently, they inherently reflect the finite spatial resolution, blur, and noise characteristics of the acquisition system. Although this approach generates lesion appearances similar to those encountered in clinical images acquired with the same system, the simulated microcalcifications may differ from the underlying lesion morphology and may not fully represent the appearance of lesions acquired using other detector technologies or imaging systems.

The restoration framework also relies on a noise model whose parameters were estimated from uniform phantom measurements acquired under controlled conditions. Although these measurements were performed using the same mammography system employed to acquire the clinical images, the estimated parameters represent an approximation of the average system behavior rather than an exact characterization of the noise in every clinical image. Variations in detector calibration, beam quality, scatter conditions, and other acquisition‐related factors may produce discrepancies between the modeled and actual noise characteristics. Because these parameters are used as inputs to the restoration pipeline, such discrepancies could affect restoration performance.

The restoration pipeline was configured to target the signal and noise characteristics predicted for an acquisition at approximately twice the original radiation dose. However, no matched higher‐dose acquisitions were available for direct comparison. Therefore, the results demonstrate improved detectability relative to the original images but do not establish equivalence to images actually acquired at the higher dose. Future phantom experiments or virtual clinical trials could evaluate this relationship directly.

Finally, the analysis was limited to simulated microcalcification clusters. Other clinically relevant findings, such as masses and architectural distortions, have different contrast, morphology, and spatial‐frequency characteristics and may respond differently to restoration. Future work should therefore evaluate the method using a broader range of simulated and clinically confirmed lesions. The framework could also be extended to DBT, including restoration of projection images and reconstructed volumes, for which correlations across projections and reconstructed slices may require additional modeling. Further investigations should also include larger multi‐institutional reader cohorts, whole mammograms, realistic lesion‐search tasks, and direct comparisons with actual or accurately simulated higher‐dose acquisitions to determine the generalizability and clinical relevance of the observed improvement.

## 5 CONCLUSIONS

In conclusion, the proposed extended MB restoration pipeline improved the detectability of simulated microcalcification clusters under the controlled 4AFC conditions investigated. The primary GLMM indicated higher odds of a correct response for restored images than for original images (OR = 1.57, 95% CI: 1.15–2.14, p=0.005). By incorporating an extended noise model, variance stabilization, and a filtering strategy that explicitly accounts for spatial noise correlation, the pipeline was configured to target the signal and noise characteristics predicted by the model for an acquisition at approximately twice the original radiation dose, without requiring additional radiation exposure. These findings suggest that advanced MB restoration techniques represent a promising strategy for enhancing mammographic image quality and supporting breast radiologists in lesion‐detection tasks. Further studies should determine whether the observed improvement translates into improved diagnostic performance under more realistic reading conditions, using whole mammograms rather than cropped ROIs, clinically confirmed lesions, and observer paradigms that more closely reflect clinical interpretation, including ROC analysis and free‐response ROC lesion‐search tasks.

## CONFLICT OF INTEREST STATEMENT

The authors declare no conflicts of interest.

## ETHICS STATEMENT

The retrospective use of anonymized clinical mammography images was approved by the Research Ethics Committee of Barretos Cancer Hospital (CAAE #78625417.1.1001.5437).

## Data Availability

The complete restoration framework and the MATLAB implementation are publicly available in the LAVI‐USP GitHub repository: https://github.com/LAVI‐USP/MedPhy2026_MB_Image_Restoration The repository facilitates the application of the proposed framework to other studies and image datasets. The clinical images used in this study are not publicly available due to data‐sharing restrictions.

## Footnotes

1 Note that, in this work, $\xi_{s}$ and $\xi_{e}$ denote the standard deviations of the structural and electronic noise components, whereas in^36^ these variables denote the corresponding variances.
